# Novel *APC* promoter and exon 1B deletion and allelic silencing in three mutation-negative classic familial adenomatous polyposis families

**DOI:** 10.1186/s13073-015-0148-0

**Published:** 2015-05-04

**Authors:** Yiing Lin, Shin Lin, Melanie D Baxter, Lawrence Lin, Susan M Kennedy, Zhengyan Zhang, Paul J Goodfellow, William C Chapman, Nicholas O Davidson

**Affiliations:** Department of Surgery, Washington University School of Medicine, St. Louis, Missouri USA; Department of Cardiovascular Medicine, Stanford University, Palo Alto, California USA; St. Francis Medical Center, Cape Girardeau, Missouri USA; College of Medicine, Ohio State University, Columbus, Ohio USA; Division of Gastroenterology, Washington University School of Medicine, 660 S. Euclid Ave, Campus Box 8124, St. Louis, Missouri MO 63110 USA

## Abstract

**Background:**

The overwhelming majority (approximately 80%) of individuals with classic familial adenomatous polyposis (FAP) exhibit mutations in the coding sequence of the adenomatous polyposis coli (*APC*) tumor suppressor gene. Families without detectable *APC* mutations are unable to benefit from the use of genetic testing for clinical management of this autosomal dominant syndrome.

**Methods:**

We used exome sequencing and linkage analysis, coupled with second-generation sequencing of the *APC* locus including non-coding regions to investigate three *APC* mutation-negative classical FAP families.

**Results:**

We identified a novel ~11 kb deletion localized 44 kb upstream of the transcription start site of *APC* that encompasses the *APC* 1B promoter and exon. This deletion was present only in affected family members of one kindred with classical FAP. Furthermore, this same deletion with identical breakpoints was found in the probands of two additional *APC* mutation-negative classical FAP kindreds. Phasing analysis of single nucleotide polymorphisms (SNPs) around the deletion site in the three probands showed evidence of a shared haplotype, suggesting a common founder deletion in the three kindreds. SNP analysis within the coding sequence of *APC*, revealed that this ~11 kb deletion was accompanied by silencing of one of the *APC* alleles in blood-derived RNA of affected individuals.

**Conclusions:**

These results support the causal role of a novel promoter deletion in FAP and suggest that non-coding deletions, identifiable using second-generation sequencing methods, may account for a significant fraction of *APC* mutation-negative classical FAP families.

## Background

Familial adenomatous polyposis (FAP) is an inherited, autosomal dominant syndrome characterized by the development of colonic polyposis and cancer progression by 40 years of age when left untreated. The classical FAP phenotype results in the growth of hundreds to thousands of adenomas, caused in approximately 70% to 80% of affected families by germline mutations in the tumor suppressor gene adenomatous polyposis coli (*APC*) on chromosome 5q21-22 [1,2]. An attenuated FAP phenotype results in generally fewer than 100 adenomas, and fewer than 10% of such families have detectable *APC* mutations [1]. Mutations in the gene Mut Y homolog (*MUTYH*) have been associated with an autosomal recessive form of inherited colonic polyposis and are not observed in classical or attenuated FAP [3].

Hundreds of pathologic mutations in *APC* have been described which result in FAP, most by producing a truncated protein product [4]. Such mutations occur throughout the 15 exons of *APC*, although mutation hot spots occur between codons 1,000 to 1,500 of exon 15 [2,3]. Routine clinical genetic testing has therefore consisted of Sanger sequencing of the 15 exons of *APC* as well as southern blot analysis on all 15 exons to detect rearrangements or copy number variations [5]. The inability to determine a causal mutation of FAP in ‘*APC* mutation-negative’ families precludes the identification of unaffected members and necessitates intensive colonic surveillance of all individuals of the family [5].

Reports have begun to describe mutations surrounding exon 1B of *APC* in mutation-negative families: A ~61 kb deletion which partially overlapped promoter 1B was described in a Swedish FAP family [6]. A large deletion containing promoter 1A was found in a Mennonite family through multiplex ligation-dependent probe amplification, but the genomic breakpoint coordinates were not defined [7]. A ~21 kb deletion containing promoter 1B was described in the proband and affected father of a Bulgarian family [8]. More recently, distinct promoter deletions were identified in *APC* in mutation-negative families which were approximately 34 kb [9] and approximately 132 kb [10] in size. These deletions are particularly relevant given prior observations that one *APC* allele is silenced in many individuals with *APC* mutation-negative FAP [11].

Here we investigated a multi-generational *APC* mutation-negative FAP kindred, using second-generation sequencing techniques that identified an ~11 kb deletion which encompasses exon 1B and the associated promoter [12]. Furthermore, we found this deletion in two additional FAP kindreds previously classified as *APC* mutation-negative by standard genetic testing. In the probands of all three kindreds, we demonstrate the absence of a transcript from one *APC* allele indicating that the ~11 kb deletion likely silences *APC* expression. These results highlight the importance of second-generation sequencing approaches to test for potentially pathologic mutations of *APC* which cannot be detected by current standard genetic tests.

## Methods

### Study subjects

Within our FAP registry, we identified three *APC* mutation-negative FAP kindreds with classical phenotypes. Participants from these kindreds gave written informed consent for participation in a study approved by the Institutional Review Board of Washington University, St. Louis (IRB# 201105464) and which conforms to the Helsinki Declaration. These kindreds were concentrated in three different states: Missouri, Illinois, and Idaho, and were not known to be related. The probands from each kindred underwent clinical genetic testing with Colaris AP® (Myriad Genetic Laboratories, Salt Lake City, UT, USA), which sequences 15 exons of *APC* and exons 7 and 13 of *MUTYH* to detect the mutations Y165C and G382D. Southern blot analysis on all exons of *APC* is also performed to detect large rearrangements. No known pathologic mutations in *APC* and *MUTYH* were detected by this method in the probands of the three kindreds.

Blood from participants was collected in tubes without additives for DNA extraction and in PAXgene Blood RNA Tubes (PreAnalytiX; Qiagen, Valencia, CA, USA) for RNA preservation and extraction. DNA blood extraction was performed using the Gentra Puregene Blood Kit (Qiagen), and RNA blood extraction was performed using the PAXgene Blood RNA Kit (Qiagen).

### Exome sequencing and linkage analysis

Exome capture was performed with the SureSelect 38 Mb All Exon kit (Agilent, Salta Clara, CA, USA) for members of kindred 0124 III-5, III-3, III-4, and the SureSelect 72 Mb (V4 + UTR) kit was used for individuals 0124 IV-4, V-6, IV-2, V-1, and IV-3. Paired-end sequencing (2 × 101 bp) was performed on the HiSeq 2000 platform (Illumina, San Diego, CA, USA). The sequences were aligned against the GRCh37/hg19 human reference genome using Novoalign (Novocraft, Selangor, Malaysia), optical duplicate sequence removal was performed using samtools [13], and variant calls were identified using samtools and GATK [14]. Local realignment and base quality recalibration using GATK were performed prior to variant calling, and a genotype quality GQ threshold of 20 was used to retain single nucleotide variants (SNVs) for further analysis. All reported genomic coordinates are in relation to the GRCh37/hg19 reference human genome. The sequencing data is available at dbGaP with accession number phs000904.v1.p1.

To search for candidate exon mutations which segregated with the FAP phenotype in kindred 0124, we applied a series of filters to the identified SNVs. We required that a candidate SNV follow an autosomal pattern of inheritance and retained only those which were heterozygous in affected individuals and homozygous in unaffected ones. The SNVs were annotated for function using ANNOVAR [15], and only non-synonymous mutations were retained. Finally, single nucleotide polymorphisms (SNPs) in dbSNP132 [16] and identified in the 1000 Genomes project [17] were removed. To perform linkage analysis, the unfiltered, aggregated SNVs were analyzed with MERLIN using parametric linkage analysis with a rare dominant disease model [18].

### Coloseq™ testing and deletion validation

Subjects IV-4 of kindred 0124, IV-1 of kindred 0130, and III-2 of kindred 0163 underwent sequencing of the *APC* locus with the ColoSeq™ Polyposis Panel, which performs second-generation sequencing of *APC* and *MUTYH* to cover all exons, introns and flanking sequences [12]. An ~11 kb deletion encompassing exon and promoter 1B was detected in all three individuals between coordinates chr5:112,034,824-112,045,845. To verify the presence of this deletion, PCR using standard conditions and primers P1, P2, and P3 (Table 1) were used. In the presence of template genomic DNA with the ~11 kb deletion, primers P1 and P3 form a 298 bp PCR product, whereas primers P1 and P2 do not result in a product because the binding site for primer P2 is absent. With a normal genomic DNA template, primers P1 and P2 form a 373 bp product, whereas primers P1 and P3 do not form a product because the distance of their binding sites exceeds 11 kb. The PCR amplicons were sequenced with Sanger chemistry using the ABI BigDye Terminator Mix (version 3, Applied Biosystems, Foster City, CA, USA).

**Table 1:** **Primer sequences and coordinates**

**Primer name**	**Primer sequence**	**Left-most position of primer**
		**(GRCh37/hg19 coordinates)**
P1	5′ CTAGGCTATACCATCTAGGCTTGTG 3′	chr5:112,034,609
P2	5′ GAGGCCAGTATTACTTTGATACCC 3′	chr5:112,034,958
P3	5′ CTGACCAATATCCTCACATAGCTG 3′	chr5:112,045,903
P4	5′ GAGTTCCCTGGTGTAAATGCTCT 3′	chr5:112,032,220
P5	5′ CCCTAACAACCAGTCTGAGTAGC 3′	chr5:112,032,727
P6	5′ AGTAAACCTGGAAAGAGCACCAC 3′	chr5:112,032,926
P7	5′ CTTCTAGTACTTAAAGGGACAGCACC 3′	chr5:112,033,107
P8	5′ ATCCTCCATATCTGTGGGTTCTG 3′	chr5:112,034,220
P9	5′ GCTCTGCTTAACGACAGGAATAC 3′	chr5:112,034,692
P10	5′ GTAGAGGCCAGATACACTAATGTCC 3′	chr5:112,049,943
P11	5′ CTACTATGCCAGACAGTATTCCAGAC 3′	chr5:112,050,557
P12	5′ CCAGAGATCAGTAGTCTTTCCAGAG 3′	chr5:112,051,725
P13	5′ CAGAGACCTGCACTCAATAAATGG 3′	chr5:112,052,217
P14	5′ ACAGTGTCTGGCTCTCTGAGATACT 3′	chr5:112,029,655
P15	5′ CATCTATGTAGGCTAGAGAGGGAGA 3′	chr5:112,046,239
P16	5′ AGTTAGCTGCTGGAGAAGGAGTTAG 3′	chr5:112,176,294
P17	5′ GCTGGTAACTTTAGCCTCTGATTC 3′	chr5:112,176,956
P18	5′ GAATCAGAGGCTAAAGTTACCAGC 3′	chr5:112,176,956
P19	5′ GTCACTGAGAGAACTCAGAGAGGAA 3′	chr5:112,177,189

### SNP genotyping and haplotype phasing

To query SNPs flanking the promoter deletion, the following primer pairs were used (Table 1): primers P4/P5 for rs76768628, rs60905866, and rs10463643; primers P6/P7 for rs10051624; primers P8/P9 for rs2900066, rs6594643, and rs6594644; primers P10/P11 for rs4705559 and rs7704618; and primers P12/P13 for rs1661035 and rs76028897. To amplify DNA specifically from the chromosomal strand harboring the ~11 kb deletion, two primer pairs were used: P8/P13 and P14/P15 (Table 1). On chromosomes with the ~11 kb deletion, these primer pairs resulted in amplicons of 5,541 bp and 6,956 bp in size, respectively. Because these amplicons represent DNA amplified only from the chromosomal strand with the ~11 kb deletion, all of the SNP genotypes determined from these amplicons were used as templates for Sanger sequencing (and haplotype phasing) using primers P4, P6, P8, P10, and P12 (Table 1).

### Allelic expression assessment

dbSNP was searched for SNPs residing in the coding region of *APC*, and rs459552 and rs465899 were found to be heterozygous in the exome sequence data of one or more individuals of kindred 0124. After extraction of RNA from blood collected in PAXgene Blood RNA Tubes, reverse transcription was performed using SuperScript III Reverse Transcriptase (Invitrogen, Grand Island, NY, USA). The following primers were used to perform PCR and subsequent sequencing for SNP assays (Table 1): primers P16/P17 for rs459552 (producing amplicon chr5:112,176,294-112,176,979) and primers P18/P19 for rs465899 (producing amplicon chr5:112,176,956-112,177,213).

## Results

### Exome sequencing of a classical FAP family reveals a LOD peak at the APC locus

**Figure 1:**
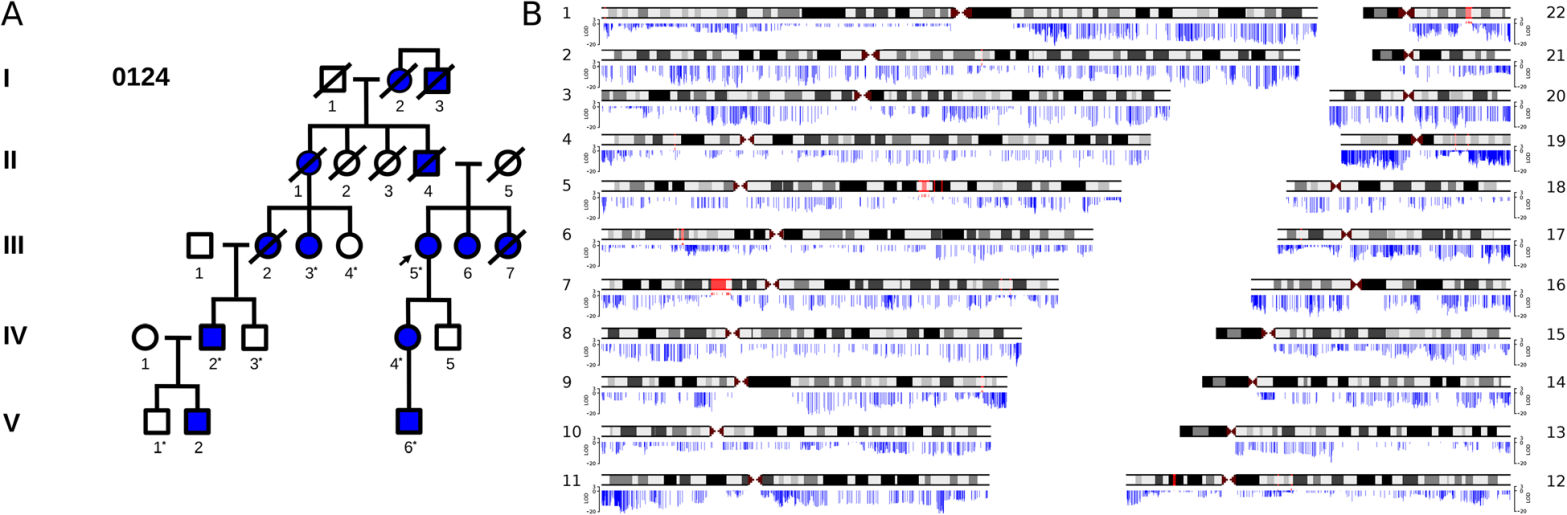
**Exome sequencing in** ***APC*** **mutation-negative kindred 0124 revealed positive LOD scores at the** ***APC*** **locus. (A)** Affected members of kindred 0124 have classical FAP phenotypes with highly-penetrant, autosomal dominant inheritance across the family. Extra-colonic malignancies were unusually common in this kindred, including duodenal adenocarcinoma (in two members), pancreas adenocarcinoma, gastric adenocarcinoma, breast cancer, leukemia, ovarian adenocarcinoma, endometrioid carcinoma, and neuroendocrine carcinoma. The proband (III-5, arrow) underwent standard genetic testing and no known FAP-causing mutation was identified. Asterisks indicate the eight individuals who underwent exome sequencing. Analysis did not reveal a credible causal mutation within the exonic sequences. Parametric linkage analysis using a rare autosomal dominant model with SNPs identified from the exome sequencing data was then performed. **(B)** LOD scores are plotted across somatic chromosomes, with positive LOD score regions marked in red and negative scores marked in blue. The maximum LOD of 2.408 occurred at 5q22, which contains the *APC* locus.

Kindred 0124 is a multi-generational family with a classical FAP phenotype (Figure 1A), including the presence of multiple extra-colonic malignancies but with no mutations in *APC* using standard genetic testing. To investigate the causal mutation accounting for FAP, we performed exome sequencing on eight individuals spanning three generations of the 0124 kindred. Analysis of the exome data revealed one non-synonymous heterozygous mutation which segregated in the affected family members and did not appear in dbSNP132 or an in-house exome sequence database. This was a c.G512A (p.R171Q) mutation in the gene Proline Rich Protein BstNI Subfamily 2 (*PRB2*) at coordinate chr12:11,546,500. *PRB2* is a member of a class of genes located on chromosome 12p13.2 which code for abundant proteins in salivary excretion [19]. Of note, this SNP has appeared in dbSNP as of version 135 and has a minor allele frequency of 0.009 in dbSNP 137. The significance of this non-synonymous mutation remains unclear in relation to the FAP phenotype.

Considering the possibility of a non-coding mutation accounting for the FAP phenotype, we turned to linkage analysis using SNPs derived from exome sequencing data. Using parametric linkage analysis with a dominant disease model [18], we found several loci with positive logarithm of the odds (LOD) scores, although none had a LOD score over 3 (Figure 1B). We did not find a positive LOD score around the *PRB2* locus, further indicating a lack of support for the role of *PRB2* in the FAP phenotype. However, we found two loci with a maximum LOD score of 2.408 - one at 5q22.2 (111.5 to 112.9 Mb) and another at 7p14.1 (37.2 to 43.3 Mb). 5q22.2 notably contains the *APC* gene and suggested the possibility of a non-coding *APC* mutation being causal in this FAP kindred. Because of the known relationships between *APC* and FAP, we chose to focus the remainder of this study at the *APC* locus.

### Second-generation sequencing of the *APC* locus reveals an 11 kb promoter deletion

**Figure 2:**
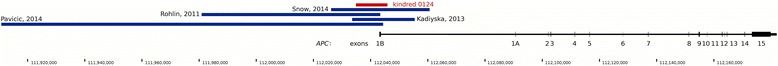
**Second-generation sequencing across the** ***APC*** **locus revealed an ~11 kb heterozygous deletion.** Affected individual IV-4 of kindred 0124 (red bar) was subjected to second-generation sequencing across the *APC* locus including the promoter, exons and introns. A heterozygous deletion approximately 44 kb upstream of *APC* exon 1A was found between coordinates chr5:112,034,824 and chr5:112,045,845 (GRCh37/hg19) which encompasses exon and promoter 1B (Genbank D13981.1). Other studies (Snow *et al.*, Rohlin *et al.*, and Kadiyska *et al.*) have reported larger promoter deletions in this region [6,8-10] (blue bars).

To search for a non-coding mutation at the *APC* locus of the affected individual IV-4 of kindred 0124, we used the ColoSeq™ assay to capture and sequence exonic, intronic, and promoter regions of the *APC* gene [12]. This assay identified an ~11 kb heterozygous deletion 44 kb upstream of the first exon of *APC* between coordinates chr5:112,034,824-112,045,845 (Figure 2). This deletion contains exon and promoter 1B (Genbank D13981.1) and represents the smallest promoter deletion currently described in an FAP family.

**Figure 3:**
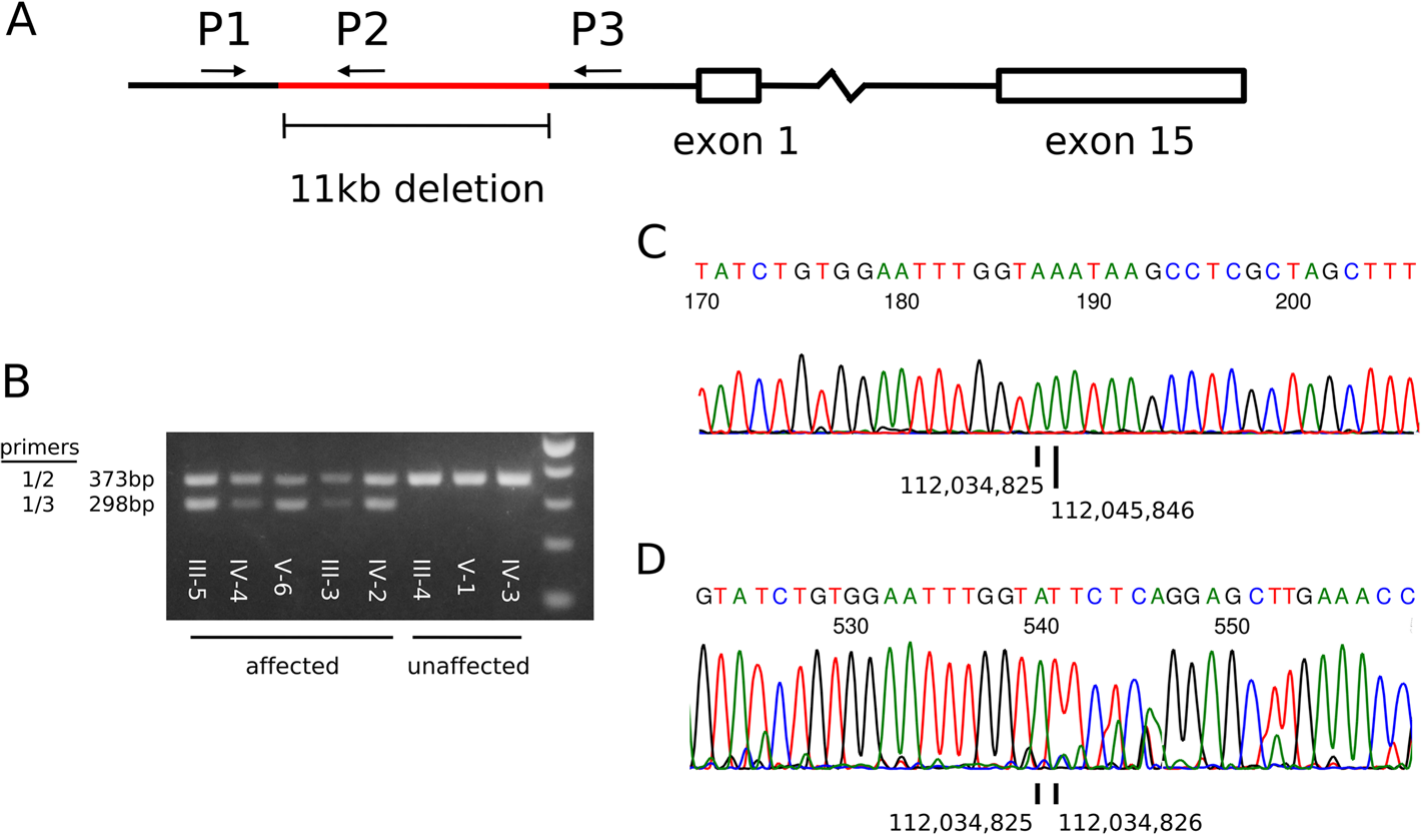
**The heterozygous ~11 kb** ***APC*** **deletion is present in all affected members of the 0124 kindred. (A)** Primers were designed to bind upstream of the ~11 kb deletion (primer 1), within the deletion (primer 2), and downstream of the deletion (primer 3). In the presence of a normal allele, primers 1 and 2 result in a 373 bp PCR product. Primers 1 and 3 do not form a product in the absence of the ~11 kb deletion but form a 298 bp PCR product when the deletion is present. **(B)** In the five affected members of kindred 0124 (III-5, IV-4, V-6, III-3, and IV-2), PCR using these primers resulted in two fragments of the expected sizes. In the three unaffected members (III-4, V-1, and IV-3), PCR produced only one fragment. **(C)** Sanger sequencing of the PCR product from affected member III-5 using primer 3 confirmed the presence of the ~11 kb deletion. The sequencing trace transitions from coordinate chr5:112,034,825 to chr5:112,045,846 (GRCh37/hg19), a span of 11,020 bp. **(D)** Sequencing the same PCR product using primer 2 showed the presence of a normal allele and the absence of the gap with a transition from coordinate chr5:112,034,825 to chr5:112,034,826.

We verified the presence of the deletion using primers designed to create a PCR product of 373 bp when a genomic template with normal *APC* promoter is present (primers 1 and 2, Figure 3A). A second primer pair creates a 298 bp PCR product only when a genomic template with the ~11 kb deletion is present (primers 1 and 3, Figure 3A). We used these primers to assess the eight members of kindred 0124 (Figure 3B). PCR in all five affected individuals generated both 298 bp and 373 bp products, indicating heterozygosity for the promoter deletion allele in *APC*. In the three unaffected individuals, only the 373 bp fragment was generated indicating the absence of the promoter deletion.

Further verifying the presence of the ~11 kb promoter deletion, we performed Sanger sequencing of the PCR products from the proband of kindred 0124. Using primer 3 for Sanger sequencing, we confirmed an 11,020 bp gap in the mapped coordinates from the sequencing trace (Figure 3C). Sanger sequencing using primer 3 also confirmed the presence of a normal allele in the proband (Figure 3D).

### 11 kb promoter deletion is found in two additional FAP kindreds

**Figure 4:**
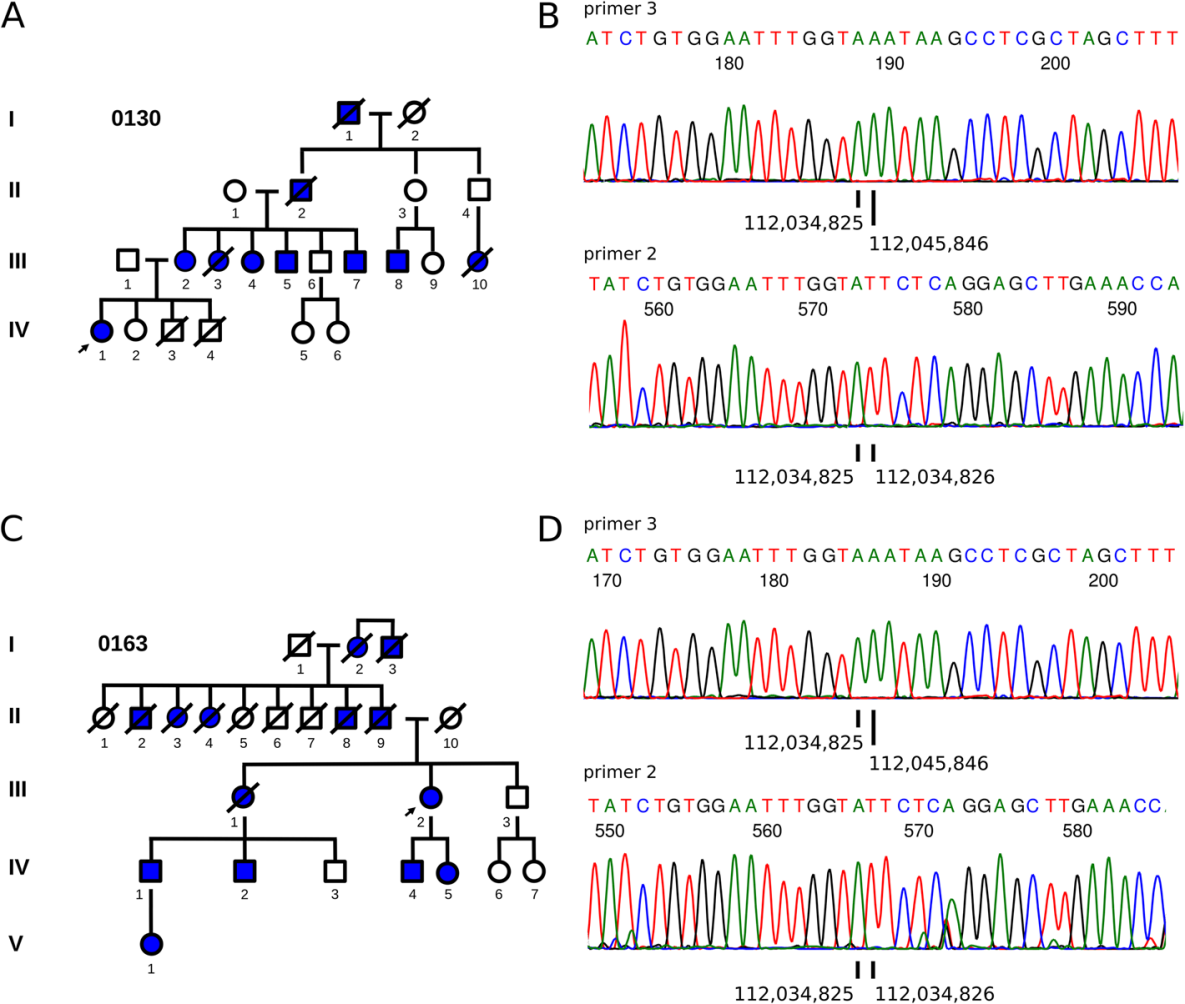
**The ~11 kb** ***APC*** **deletion is found in two other** ***APC*** **mutation-negative kindreds. (A, C)** Affected members of kindreds 0130 and 0163 have the classical FAP phenotype, and standard genetic testing of probands IV-1 in kindred 0130 and III-2 in kindred 0163 (arrows) did not identify known pathologic mutations of *APC*. Second-generation sequencing across the *APC* locus in the probands suggested that both had the same heterozygous ~11 kb *APC* deletion found in the index kindred 0124. Sanger sequencing using primer 3 confirmed the presence of the same deletion coordinates from chr5:112,034,825 to chr5:112,045,846 in the probands 0130 (IV-1) **(B)** and 0163 (III-2) **(D)**. Sequencing using primer 2 in both probands showed the presence of a normal allele as well, with contiguous coordinates from chr5:112,034,825 to chr5:112,034,826.

Two additional kindreds in our FAP registry were found to be *APC* mutation-negative by standard genetic testing. These kindreds, 0130 and 0163 (Figure 4A and C), reside in different states in America and have no known familial relationship to each other or to the index kindred 0124. To assess the presence of promoter deletions in these two additional FAP kindreds, we used the ColoSeq™ assay to sequence the *APC* locus in each proband and surprisingly found the same ~11 kb deletion. Further investigation using Sanger sequencing confirmed the identical deletion coordinates in both probands (Figure 4B and D). To exclude possible sample error, we verified those findings in a second blood sample from the probands of all three kindreds 1 year after obtaining the first samples, reinforcing our conclusions that affected members from these independent kindreds indeed harbor the identical mutation.

### Genetic relationship between kindreds revealed by haplotype phasing

**Figure 5:**
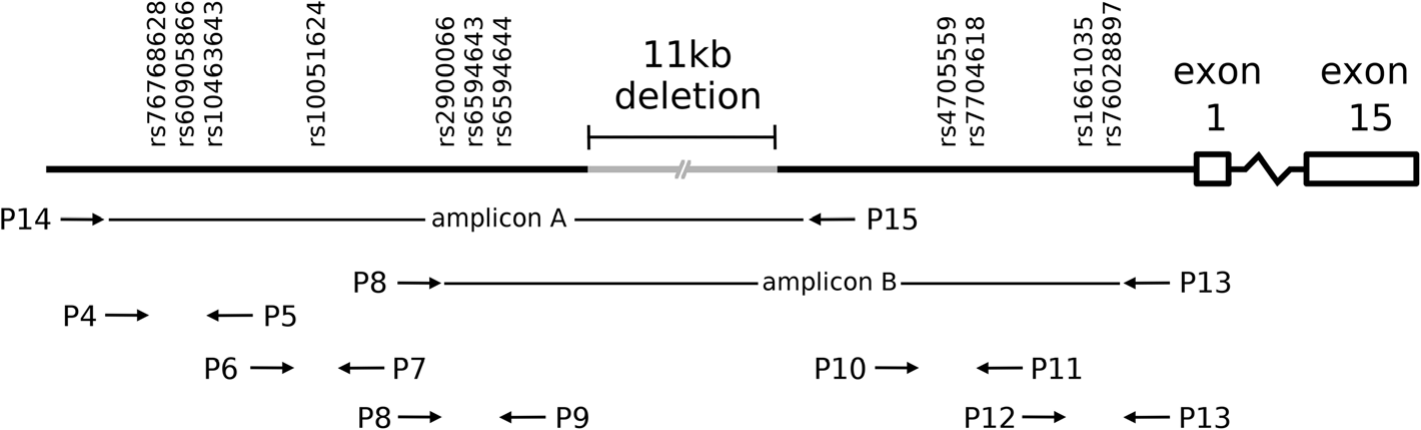
**Schematic of primer design for haplotype phasing of SNP genotypes in relation to the ~11 kb** ***APC*** **deletion.** The primer pairs P14/P15 and P8/P13 flanked the ~11 kb deletion and relevant SNPs. Only amplification of the *APC* allele with the ~11 kb deletion would result in an amplicon A size of 5.5 kb and an amplicon B size of 6.9 kb. Amplification of the allele without the ~11 kb deletion would not be efficient, as the resultant amplicons would be greater than 16 kb. Amplicons A and B were used as templates for Sanger sequencing reactions to reveal polymorphic variations in the diagrammed SNPs flanking the ~11 kb deletion.

As alluded to above, these three kindreds reside in geographically distinct regions and no known relatedness was discerned by questionnaire. Nevertheless, we examined genetic evidence of a relationship between these kindreds by determining the genetic haplotype associated with the ~11 kb deletion in each of the three probands. We first queried 11 SNPs flanking the deletion coordinates (Table 2), seven of which ranged from 229 to 2,408 bp 5′ of the deletion, and four of which ranged from 4,276 to 6,151 bp 3′ of the deletion. SNPs rs10463643, rs10051624, rs2900066, rs6594643, rs6594644, rs4705559, rs7704618, and rs1661035 were heterozygous in the probands of the 0124 and 0163 kindreds. These findings suggested that these SNPs could distinguish the haplotype associated with the ~11 kb deletion from the haplotype from the chromosomal strand without the deletion. Accordingly we phased the SNP genotypes by using a PCR strategy to selectively amplify the DNA from the chromosomal strand containing the ~11 kb deletion (Figure 5). Flanking primers were designed such that amplicons of 5.5 kb and 6.9 kb would reflect the deletion, whereas amplification from the wild type chromosomal strand would yield an amplicon 11 kb larger. Accordingly, we then isolated the 5.5 and 6.9 kb amplicons for Sanger sequencing as a result of which we were able to phase the SNP genotypes associated with the deletion. The sequencing results showed that the haplotype associated with the ~11 kb deletion was identical across the queried SNPs in each of the three probands (Table 2), providing evidence to suggest that these three kindreds share a common ancestor.

**Table 2:** **Haplotype phasing by SNP genotyping**

**SNP**	**Reference**	**Distance (bp)** ^**a**^	**0124**	**0124 phased** ^**b**^	**0130**	**0130 phased** ^**b**^	**0163**	**0163 phased** ^**b**^
SNPs 5′ to deletion								
rs76768628	A	2408	A/A	A	A/A	A	A/A	A
rs60905866	G	2250	G/G	G	G/G	G	G/G	G
rs10463643	C	2150	**C/T**	**T**	**T/T**	**T**	**C/T**	**T**
rs10051624	G	1781	**G/C**	G	G/G	G	**G/C**	G
rs2900066	A	473	**A/T**	**T**	**T/T**	**T**	**A/T**	**T**
rs6594643	C	275	**C/A**	C	C/C	C	**C/A**	C
rs6594644	T	229	**T/G**	T	T/T	T	**T/G**	T
SNPs 3′ to deletion								
rs4705559	A	4276	**A/G**	A	A/A	A	**A/G**	A
rs7704618	T	4619	**T/G**	T	T/T	T	**T/G**	T
rs1661035	G	5982	**G/C**	**C**	**C/C**	**C**	**G/C**	**C**
rs76028897	A	6151	A/A	A	A/A	A	A/A	A

^a^Distance from the adjacent breakpoint coordinate of deletion chr5:112,034,826-112,045,845.

^b^SNP typing of phased PCR amplicons.

Positions which differ from the reference base are denoted in bold.

### *APC* promoter deletion results in allelic silencing in blood

**Figure 6:**
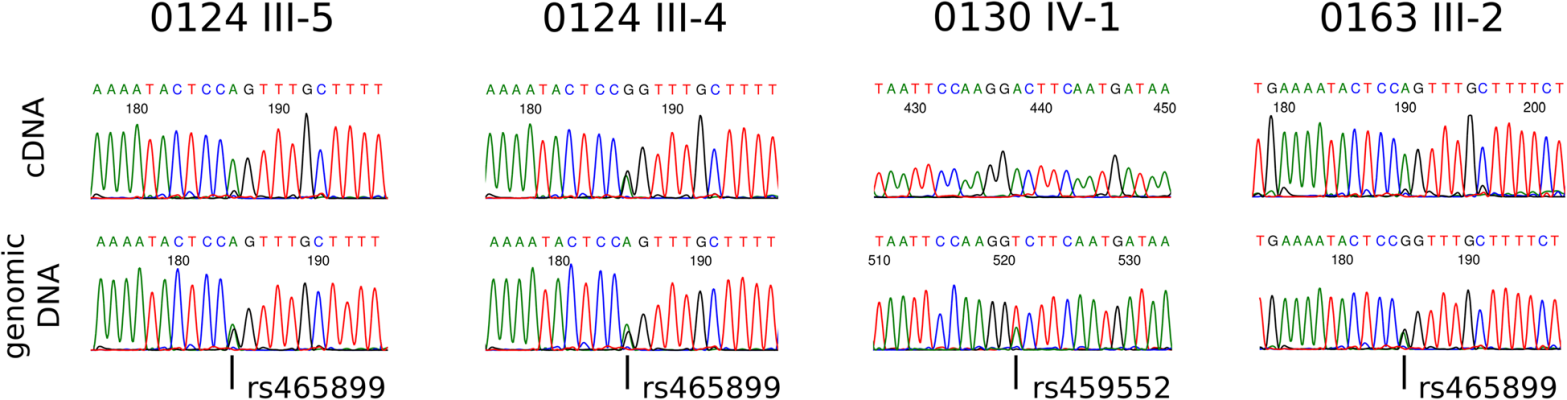
**The ~11 kb deletion silences one** ***APC*** **allele in the blood of affected individuals.** Germline heterozygous SNPs were evaluated in the RNA isolated from peripheral blood, with sample traces shown here. dbSNP rs465899 was found to be heterozygous in the proband 0124 (III-5) gDNA but not in the cDNA, whereas this SNP was heterozygous in both gDNA and cDNA in the unaffected subject 0124 (III-4). A loss of cDNA heterozygosity was also observed in proband 0130 (IV-1) with SNP rs459552, and in proband 0163 (III-2) with SNP rs465899.

*APC* is transcribed in a wide variety of tissues, including peripheral blood cells [6]. To assess the effect of the ~11 kb deletion on allelic transcription in these kindreds, we identified exonic SNPs in *APC* which were heterozygous in the germline of affected and unaffected individuals of these FAP kindreds. Silencing of an *APC* allele in individuals heterozygous for these SNPs would result in the detection of only one base at the corresponding complementary DNA (cDNA) site from expression of a single *APC* allele. Sanger sequencing of genomic DNA (gDNA) from proband III-5 of kindred 0124 showed heterozygousity at SNPs rs459552 and rs465899, but sequencing of cDNA derived from blood shows the presence of only one allele for both corresponding positions. This is in contrast to the unaffected individual III-4 of kindred 0124 whose gDNA is heterozygous for SNP rs465899, and in whom cDNA sequencing shows evidence of mRNA expression from two alleles (Table 3 and Figure 6). The silencing of one *APC* allele was also observed in affecteds from the other two kindreds with loss of cDNA heterozygosity at SNP rs459552 in proband IV-1 of kindred 0130, and SNPs rs459552 and rs465899 in proband III-2 of kindred 0163 (Table 3 and Figure 6).

**Table 3:** **Sanger sequencing evaluation of** ***APC*** **SNPs in gDNA and RNA of affected and unaffected individuals**

		**Subject**			
		**0124 (III-5)**	**0124 (III-4)**	**0130 (IV-1)**	**0163 (III-2)**
**SNP**		**Affected**	**Unaffected**	**Affected**	**Affected**
rs459552	gDNA	**A/T**	-/-	**A/T**	**A/T**
	cDNA	A/A	-/-	A/A	A/A
rs465899	gDNA	**A/G**	**A/G**	-/-	**A/G**
	cDNA	A/A	**A/G**	-/-	A/A

Sequences which are heterozygous are denoted in bold.

cDNA, complementary DNA; gDNA, genomic DNA.

## Discussion

Here we report the presence of a novel ~11 kb heterozygous deletion in the *APC* promoter of three independent *APC* mutation-negative classical FAP kindreds. Our findings add to the growing body of literature suggesting that non-coding *APC* deletions may contribute to an important fraction of *APC* mutation-negative classical FAP. The current literature is based in reports of a founder mutation in a Mennonite kindred, a kindred in the Swiss Polyposis Registry, and a single Bulgarian kindred [6-8]. However, this is the first report to our knowledge that the identical mutation exists in three classical FAP kindreds. In addition, there are informative features of the approach we used to identify this mutation that bear further discussion.

Our evaluation began with the exome sequencing of one multigenerational family with classical FAP to address the possibility that the causal mutation was a coding mutation in a novel gene. We identified a non-synonymous heterozygous mutation that segregated with the polyposis phenotype across the kindred. However, this mutation in *PRB2* remains of unclear significance since database searches revealed no plausible connection to FAP or gastrointestinal polyposis in general. Our experience parallels that of others showing some of the potential pitfalls in using exome sequencing to search for causal mutations in Mendelian diseases. For example, in the search for the causal mutation for Kabuki syndrome, Ng *et al.* performed exome sequencing on 10 unrelated affected individuals and found one rare, non-synonymous mutation (in *MUC16*) across all 10 individuals [20]. However, this particular mutation was felt to be a false positive result, and further investigations identified a more likely mutation candidate (*MLL2*).

Although exome sequencing has been used successfully to identify over 100 Mendelian disease genes [21,22], the number of unsuccessful applications of this technique is unknown. Several reasons may result in the inability to identify a mutation using exome sequencing: False negatives can result from technical issues such as poor capture and subsequent low sequence coverage of exons, or from analytical errors such as from indiscriminant candidate filtering using SNPs from public databases. Although the Agilent SureSelect Human All Exon V4 + UTR probe set captured DNA from exon 1B in our study, data from targeted capture and exome sequencing are generally not amenable to the discovery of copy number variations because of technical biases. Sequence coverage of exon 1B did not differ between affected and non-affected members of kindred 0124 (data not shown) and therefore did not reflect the ~11 kb deletion. Although the exome sequencing strategy did not yield directly a causal variant, we were able to perform linkage analysis using SNPs derived from the exome sequences. The linkage data did not yield a LOD score significant at the whole genome level, but of the positive signals identified, the one of obvious interest was at the *APC* locus.

We further investigated the *APC* locus, using a second-generation sequencing assay across the non-coding regions to reveal an ~11 kb deletion. Unlike array-based or multiplex ligation-dependent probe amplification (MLPA) techniques used to search for regions of copy number variation, the second-generation sequencing assay was able to identify the exact breakpoint coordinates. These were further confirmed by an orthogonal sequencing modality with Sanger chemistry. Presumably, another benefit of the second-generation sequencing is the ability of the technique to detect diminutive copy number variants down to insertion/deletions of a few bases in length, whereas array or MLPA techniques are able to detect only large variants greater than kilobases in length. Unexpectedly, we found the same deletion breakpoints in all three independent *APC* mutation-negative kindreds in our FAP registry. The phased typing of SNPs flanking the ~11 kb deletion further showed that the three kindreds shared the same haplotype associated with the deletion, which suggests that the deletion in these kindreds arose from a common ancestor.

This ~11 kb deletion encompasses exon and promoter 1B, and using SNP analysis on *APC* transcripts, we found that this deletion silenced one *APC* allele in affected individuals. Rolin *et al.* described a ~61 kb heterozygous deletion which resulted in a reduction in expression, but not silencing, of an *APC* allele [6]. This ~61 kb deletion removes a portion of the exon 1B 5′ untranslated region but does affect an open reading frame within it (RefSeq NM_001127511.2). The ~11 kb deletion described in this study, by contrast, deletes exon1B and its upstream sequences in its entirety. It may be that the deletion of the exon 1B 5′ untranslated region and/or its open reading frame results in its targeted degradation. Interestingly however, a ~21 kb deletion which encompasses the ~11 kb deletion described in this study was reported to reduce, but not to silence, allelic expression of *APC* [8]. The interaction of 1A and 1B promoters in different tissue types and other epigenetic regulatory mechanisms need further investigation. For example, although our results show that the ~11 kb deletion results in diminished transcription of *APC* in blood, differential promoter use may still result in *APC* transcript production using promoter 1A in other tissues.

The allelic silencing of *APC* in mutation-negative kindreds is consistent with observations made over a decade ago. Using a method of isolating individual chromosomes from affected individuals in hybrid rodent cells to perform monoallelic mutation analysis, one *APC* allele was found to be silenced in seven of nine FAP subjects [11]. The mechanism of allelic silencing could not be determined at that time, however.

## Conclusions

We provide evidence that an ~11 kb deletion encompassing exon and promoter 1B of *APC* which segregates with the FAP phenotype in three affected kindreds results in allelic silencing. Second-generation sequencing techniques is ideal for the detection of such deletions, as they can vary greatly by size and position (Figure 2). Furthermore, the availability of such assays in the clinical setting such as with ColoSeq™ has the potential to readily impact the clinical management of FAP kindreds.

## Abbreviations

APC: Adenomatous polyposis coli
FAP: Familial adenomatous polyposis
LOD: Logarithm of the odds
